# Effect of oral sodium bicarbonate on fibroblast growth factor-23 in patients with chronic kidney disease: a pilot study

**DOI:** 10.1186/s12882-016-0331-6

**Published:** 2016-08-05

**Authors:** Wei Chen, Michal L. Melamed, Thomas H. Hostetter, Carolyn Bauer, Amanda C. Raff, Anthony L. Almudevar, Amy Lalonde, Susan Messing, Matthew K. Abramowitz

**Affiliations:** 1 Department of Medicine, University of Rochester School of Medicine and Dentistry, 601 Elmwood Avenue, Box 675, Rochester, NY 14642 USA; 2 Department of Biostatistics & Computation Biology, University of Rochester School of Medicine and Dentistry, Rochester, NY USA; 3 Department of Medicine, Albert Einstein College of Medicine, Bronx, NY USA; 4 Department of Epidemiology & Population Health, Albert Einstein College of Medicine, Bronx, NY USA; 5 Department of Medicine, Case Western Reserve University School of Medicine, Cleveland, OH USA

**Keywords:** Metabolic acidosis, Fibroblast growth factor-23, Sodium bicarbonate, Alkali therapy, Mineral metabolism, Vitamin D

## Abstract

**Background:**

The regulation of fibroblast growth factor-23 (FGF23) secretion in patients with chronic kidney disease (CKD) is incompletely understood. An *in vitro* study showed that metabolic acidosis increased FGF23 in mouse bone. The objective of this study is to evaluate the effect of oral sodium bicarbonate on circulating FGF23 levels in patients with CKD.

**Methods:**

This was a single-blind pilot study. Twenty adults with estimated glomerular filtration rate between 15–45 mL/min/1.73 m^2^ and serum bicarbonate between 20–24 mEq/L were treated with placebo for 2 weeks, followed by increasing doses of oral sodium bicarbonate (0.3, 0.6 and 1.0 mEq/kg/day) in 2 week intervals for a total of 6 weeks. C-terminal FGF23 levels were measured at the initial visit, after 2 weeks of placebo and after 6 weeks of bicarbonate therapy. Wilcoxon matched-pairs signed-rank test was used to compare FGF23 before and after sodium bicarbonate.

**Results:**

After 6 weeks of oral sodium bicarbonate, the median FGF23 increased significantly from 150.9 RU/mL (IQR 107.7–267.43) to 191.4 RU/mL (IQR 132.6–316.9) (*p* = 0.048) and this persisted after excluding participants who received activated vitamin D.

**Conclusions:**

FGF23 increased after short-term oral sodium bicarbonate therapy in patients with CKD and mild metabolic acidosis. It is unclear whether this was due to the alkalinizing effect of sodium bicarbonate or other factors.

**Trial registration:**

The study was registered at ClinicalTrials.gov (NCT00888290) on April 23, 2009.

## Background

Fibroblast growth factor 23 (FGF23) is a hormone produced in osteocytes and osteoblasts [1–3]. Its primary physiological actions are to induce phosphaturia by down-regulating the type IIA sodium-phosphate co-transporter in the proximal tubule, reduce systemic 1,25-dihydroxyvitamin D (1,25-OH vitamin D) production and inhibit parathyroid hormone (PTH) secretion [4–7]. Elevated FGF23 levels are associated with multiple adverse outcomes including kidney disease progression, cardiovascular events and death [8–12]. The regulation of FGF23 secretion in patients with chronic kidney disease (CKD) is incompletely understood.

Since metabolic acidosis stimulates bone resorption and regulates osteoblast activity and osteoblasts produce FGF23, metabolic acidosis may regulate FGF23 secretion [13]. Patients with CKD develop chronic metabolic acidosis due to loss of functioning renal mass and inability to excrete the acids that are generated through metabolism of dietary acid precursors. An *in vitro* study demonstrated that metabolic acidosis increased FGF23 concentration and RNA expression in mouse bone [13]. If metabolic acidosis increases FGF23, then alkali therapy would be expected to lower FGF23 and possibly reduce mortality in patients with CKD. The effect of alkali therapy on FGF23 regulation has not been examined in human studies. The objective of this study is to evaluate the effect of alkali therapy, using oral sodium bicarbonate, on circulating FGF23 levels in patients with CKD. We hypothesized that administration of sodium bicarbonate would lower FGF23.

## Methods

### Study population and design

This was a single-blind pilot study, conducted at Montefiore Medical Center, Bronx, New York. Details of the population and design were published previously [14]. Briefly, the study included 20 adults with estimated glomerular filtration rate (eGFR) between 15–45 mL/min/1.73 m^2^ and serum bicarbonate between 20–24 mEq/L. Participants were blinded to treatment status and investigators were not. After the initial visit, participants were treated with placebo for 2 weeks, followed by increasing doses of oral sodium bicarbonate in 2 week intervals for a total of 6 weeks. The doses of sodium bicarbonate were 0.3, 0.6 and 1.0 mEq/kg per day. Blood and 24-h urine samples were collected at the initial visit and during the final 2 days of each 2-week interval. Blood and urine samples were stored at −80 °C.

### Data collection and measurement

Serum and urine chemistry values were measured by routine procedures in the clinical laboratory at Montefiore Medical Center. Serum bicarbonate was measured using the phosphoenolpyruvate carboxylase method. Serum and urine creatinine were measured by a modified kinetic Jaffe reaction. eGFR was calculated using the CKD Epidemiology Collaboration Equation [15]. Serum 25-OH vitamin D was measured by a validated liquid chromatography/tandem mass spectrometry analysis. C-terminal FGF23 was measured using enzyme-linked immunosorbent assay (Immutopics International) in the stored specimens. FGF23 was measured at the initial visit, after 2 weeks of placebo and after 6 weeks of bicarbonate therapy.

### Statistical analyses

Values of variables before sodium bicarbonate were calculated by averaging the values at the initial visit and after the 2-week placebo. To compare variables before and after sodium bicarbonate, a paired *t*-test was used if there was no violation of test assumptions, and a Wilcoxon matched-pairs signed-rank test was used if assumptions were violated. Since activated vitamin D has been shown to increase FGF23 in patients with CKD [16, 17], sensitivity analyses were done after excluding participants who were taking nutritional or activated vitamin D using Wilcoxon matched-paired signed-rank tests. To estimate the relationship of FGF23 with other variables, a linear mixed model was used. Graphical inspection of the data suggested a logarithmic transformation of FGF23, and log base 2 transformation was carried out. Visit time was included as a factor to control for time variation of the response log-transformed FGF23, and a subject level random effect was included. Analyses were performed using STATA 12.1 (StataCorp, Texas) except the linear mixed models, which were fit using the R function linear mixed-effect (Venables, W.N. and Ripley, B.D (2002) “Modern Applied Statistics with S”, 4^th^ Edition, Springer-Verlag). A *p*-value <0.05 was considered statistically significant.

## Results

### Participant baseline characteristics

**Fig. 1:**
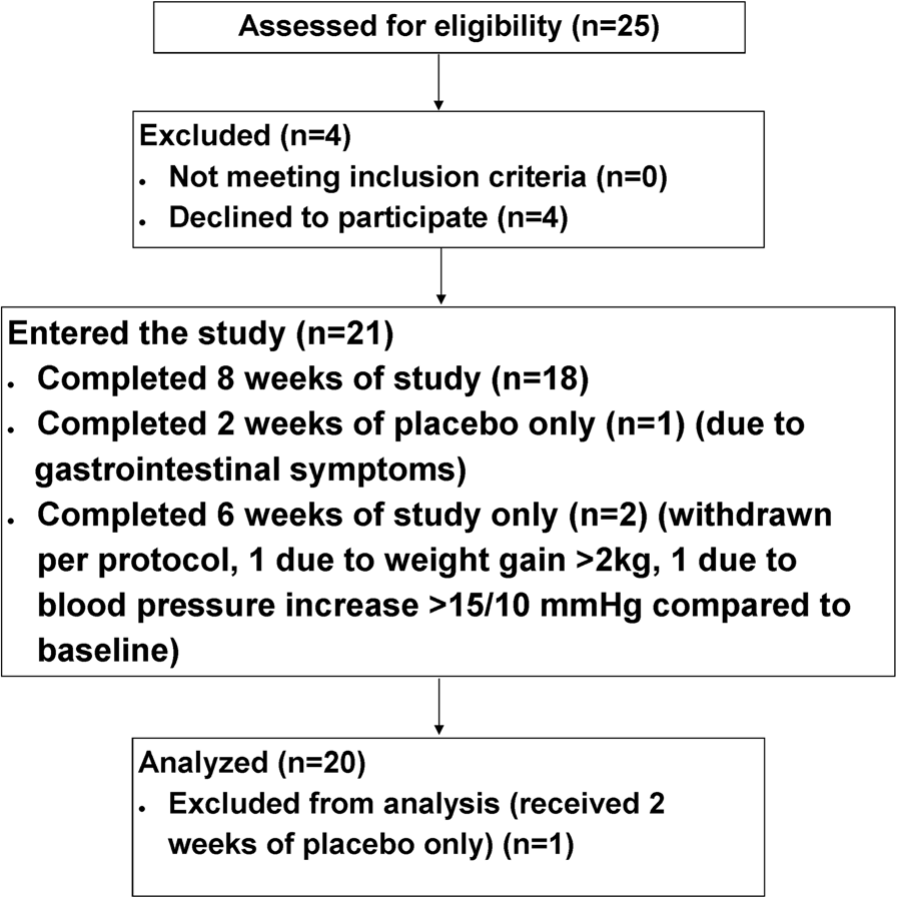
Enrollment. Twenty participants were included in the final analysis. Two completed only 6 weeks of the study and received 2 weeks of placebo and 4 weeks of sodium bicarbonate

Enrollment is shown in Fig. 1. Baseline characteristics of participants are listed in Table 1. The mean age was 63 ± 11 years. Twelve participants (60 %) were female. Participants were either black or Hispanic. Almost all had diagnoses of diabetes mellitus or hypertension. The mean eGFR was 32.9 ± 8.9 ml/min/1.73 m^2^ with mean bicarbonate of 23.0 ± 2.4 mEq/L. The median FGF23 was 157.4 RU/mL (Interquartile range [IQR] 90.2-289.4). Nine participants were receiving vitamin D at baseline (nutritional vitamin D—ergocalciferol/cholecalciferol [*n* = 6] or paricalcitol [*n* = 3]; no other activated vitamin D were prescribed). Among them, one participant (participant #3) had paricalcitol dose increased during the study, and one participant (participant #16) had very high FGF23 at baseline (824.9 RU/mL). None of the participants received phosphate binders during the study period. Fifteen participants were receiving diuretics. Two of them had diuretic dose increased prior to their last visit while others remained on the same dose throughout the study.

**Table 1:** Baseline characteristics of participants (*n* = 20)

Characteristic	Value
Age (year)	63 ± 11
Female No. (%)	12 (60)
Race/ethnicity No. (%)	
White	0 (0)
Black	11 (55)
Hispanic	9 (45)
Others	0 (0)
Blood pressure (mmHg)	134/70 ± 13/7
eGFR (ml/min per 1.73 m^2^)	32.9 ± 8.9
Serum bicarbonate (mEq/L)	23.0 ± 2.4
FGF23 (RU/mL)	157.4 (90.2–289.4)
25-OH vitamin D (ng/mL)	36 ± 13

Age, blood pressure, eGFR, serum bicarbonate, 25-OHvitamin D were expressed as mean ± standard deviation. FGF23 was expressed as median (interquartile range)

Abbreviations: *eGFR* estimated glomerular filtration rate, *FGF23* fibroblast growth factor-23, *RU* rack unit, *25-OH vitamin D* 25-hydroxyvitamin D

### Effect of sodium bicarbonate therapy

After 2-weeks of placebo, there was no significant change in bicarbonate or FGF23 compared to the initial visit (Table 2). Therefore, bicarbonate and FGF23 values before sodium bicarbonate were calculated by averaging the values at the initial visit and after 2-weeks of placebo. Eighteen participants received 6 weeks of sodium bicarbonate, and 2 participants were withdrawn from the study after receiving 4 weeks of active medication (Fig. 1). After sodium bicarbonate therapy, there was no change in eGFR. The mean bicarbonate increased from 22.6 ± 2.3 mEq/L to 25.5 ± 2.2 mEq/L (*p* < 0.001). No statistically significant changes were observed in 25-OH vitamin D, 1,25-OH vitamin D, serum calcium, serum phosphate, urine calcium, urine phosphate, urine sodium, fractional excretion of phosphate or sodium although the *p*-value was marginally significant for urine sodium (*p* = 0.07) (Table 2).

**Table 2:** Effect of sodium bicarbonate therapy

	Initial	2-week placebo	*p*-value
Serum Bicarbonate (mEq/L)	23.0 ± 2.3	22.2 ± 2.8	0.17
FGF23 (RU/mL)	157.4 (90.2–289.4)	161.7 (104.5–245.5)	0.91
	Before sodium bicarbonate^a^	After sodium bicarbonate	*p*-value
Systolic blood pressure (mmHg)	131 ± 11	130 ± 14	0.71
Diastolic blood pressure (mmHg)	69 ± 8	70 ± 10	0.66
Serum Bicarbonate (mEq/L)	22.6 ± 2.3	25.5 ± 2.2	< 0.001
eGFR (ml/min per 1.73 m^2^)	40.6 ± 15.6	40.0 ± 15.1	0.65
FGF23 (RU/mL)	150.9 (107.7–267.43)	191.4 (132.6–316.9)	0.048
25-OH vitamin D (ng/mL)	36 ± 13	37 ± 14	0.60
1, 25-OH vitamin D (pg/mL)^c^	42 (21–58)	42 (18–57)	0.79
Serum calcium (mg/dL)	9.9 ± 0.5	9.9 ± 0.5	0.39
Serum phosphate (mg/dL)	4.0 ± 0.8	3.9 ± 0.8	0.31
Urine calcium (mg/mg Cr)^b^	0.03 (0.02–0.04)	0.01 (0.01–0.05)	0.09
Urine phosphate (mg/mg Cr)^b^	0.47 (0.28–0.61)	0.42 (0.32–0.55)	0.84
Urine sodium (mg/mg Cr)^b^	2.4 ± 0.7	2.8 ± 1.0	0.07
Fractional excretion of phosphate (%)	18 (14–24)	21 (15–26)	0.22
Fractional excretion of sodium (%)	1.4 (1.1–2.4)	1.6 (1.1–2.2)	0.44

Serum bicarbonate, 25-OH vitamin D, serum calcium, phosphate and urine sodium were expressed as mean ± standard deviation. FGF23, 1,25-OH vitamin D, urine calcium and phosphate, fractional excretion of phosphate and sodium were expressed in median (interquartile range)

Abbreviations: *eGFR* estimated glomerular filtration rate, *25-OH vitamin D* 25-hydroxyvitamin D, *1,25-OH vitamin D* 1,25-dihydroxyvitamin D, *FGF23* fibroblast growth factor-23

^a^Values of variables for “before sodium bicarbonate” were the mean of values between the initial visit and after 2-week placebo

^b^urine calcium, phosphate and sodium were normalized by dividing urine creatinine. The mean urinary sodium increased from 2.4 ± 0.7 to 2.6 ± 0.9 mg/mg Cr after excluding 2 participants who had their diuretics dose increased prior to the last visit (*p* = 0.20)

^c^Only 11 participants had 1,25-OHvitamin D level available at before and after 6-week sodium bicarbonate therapy

**Fig. 2:**
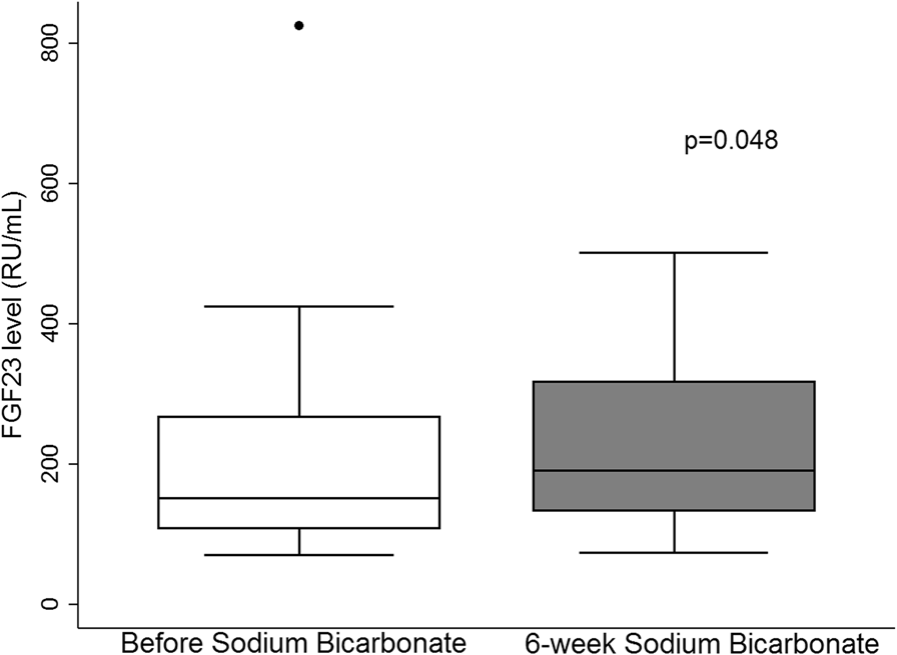
FGF23 before and after sodium bicarbonate therapy (*n* = 20). FGF23 before sodium bicarbonate therapy was calculated by averaging FGF23 at the initial visit and after 2-week placebo. Participant #16 was the outlier

FGF23 increased in 14 (70 %) participants. The median FGF23 increased significantly from 150.9 RU/mL (IQR 107.7–267.43) to 191.4 RU/mL (IQR 132.6–316.9) (*p* = 0.048) (Fig. 2). This finding remained statistically significant after excluding the 3 participants that were on paricalcitol (*p* = 0.009) or after excluding participant #16, who had a very high FGF23 at baseline (*p* = 0.01). The median FGF23 increased after excluding all 9 participants on vitamin D or after excluding participant #3, who had an increased paricalcitol dose during the study, but it was not statistically significant (*p* = 0.08 for both) (Table 3).

**Table 3:** Sensitivity analysis after excluding participants on vitamin D

FGF23 (RU/mL)	Before sodium bicarbonate^a^	After sodium bicarbonate	*p*-value
Taking vitamin D^b^			
Yes (*n* = 9)	217.0 (146.4–353.2)^*^	327.4 (231.7–410.9)	0.31
No (*n* = 20)	121.2 (85.1–162.4)^*^	147.4 (107.0–196.5)	0.08
Taking paricalcitol			
Yes (*n* = 3)	353.2 (98.9–824.9)^**^	399.1 (327.4–410.9)	0.59
No (*n* = 17)	146.4 (116.5–217.0)^**^	185.7 (129.9–238.4)	0.009
Increased paricalcitol dose			
Yes (*n* = 1)^c^	98.8	399.1	N/A
No (*n* = 19)	155.5 (116.5–286.5)	186.4 (129.9–306.4)	0.08
Participant #16^d^			
Yes (*n* = 1)	824.9	410.9	N/A
No (*n* = 19)	146.4 (98.8–248.3)	186.4 (129.9–306.4)	0.01

FGF23 was expressed in median (interquartile range)

Abbreviation: *N/A* not applicable

^*^ *p*-value comparing FGF23 between those taking vitamin D and those not taking vitamin D = 0.06

^**^ *p*-value comparing FGF23 between those taking paricalcitol and those not taking paricalcitol = 0.02

^a^Variables for “before sodium bicarbonate” were the mean of values between the initial visit and after 2-week placebo

^b^Vitamin D used included ergocalciferol, cholecalciferol and paricalcitol

^c^Participant #3 had paricalcitol dose increased during the study

^d^Participant #16 had very a high FGF23 level at baseline

### Bivariate associations with FGF23

There was no significant association between bicarbonate and FGF23 (log-transformed) (regression coefficient = −0.032, *p* = 0.21). There was no significant association of FGF23 with 25-OH vitamin D, serum calcium, urine calcium or urine sodium. FGF23 was inversely associated with 1,25-OH vitamin D (*p* = 0.005), and positively associated with serum and urine phosphate (*p* = 0.001, 0.02 respectively) (Table 4).

**Table 4:** Bivariate Association with log-transformed FGF23 (RU/mL)

	Regression Coefficient	*p*-value
Serum Bicarbonate (mEq/L)	-0.032	0.21
25-OH vitamin D (ng/mL)	-0.005	0.45
1, 25-OH vitamin D (pg/mL)	-0.012	0.005
Serum calcium (mg/dL)	0.032	0.83
Serum phosphate (mg/dL)	0.282	0.001
Urine calcium (mg/mg Cr)	2.911	0.34
Urine phosphate (mg/mg Cr)	1.274	0.02
Urine sodium (mg/mg Cr)	0.131	0.22

Urine calcium, phosphate and sodium were normalized by dividing by urine creatinine

Abbreviations: *FGF23* fibroblast growth factor-23, *RU* rack unit, *25-OH vitamin D* 25-hydroxyvitamin D1, *25-OH vitamin D* 1,25-dihydroxyvitamin D

## Discussion

This is the first human study evaluating the effect of oral sodium bicarbonate on the regulation of circulating FGF23. We found that oral sodium bicarbonate did not decrease FGF23 in patients with CKD and mild acidosis. Contrary to our hypothesis, there was a statistically significant increase in C-terminal FGF23 levels after 6 weeks of oral sodium bicarbonate (Fig. 2), and this effect persisted after excluding participants who received activated vitamin D (Table 3).

The FGF23 levels were similar to the levels reported by others. The median C-terminal FGF23 was 157.4 RU/mL (IQR 90.2–289.4) with a mean eGFR of 32.9 ± 8.9 ml/min/1.73 m^2^ at the initial visit. In the Chronic Renal Insufficiency Cohort (CRIC) study, the median C-terminal FGF23 was 145 RU/mL (IQR 96–239) in patients with a mean eGFR of 42.8 ± 13.5 ml/min/1.73 m^2^ [9]. Elevated FGF23 has been linked to several adverse clinical outcomes in patients with CKD, including kidney disease progression, cardiovascular disease and death [8–12]. In a cohort of 227 patients with CKD, FGF23 was an independent predictor of kidney disease progression after a median of 53 months follow up [10]. Isakova et al. [9] used the data from the CRIC study and demonstrated that higher FGF23 was independently associated with a greater risk of death. In our study, after 6 weeks of oral sodium bicarbonate, the median FGF23 increased significantly from 150.9 RU/mL to 191.4 RU/mL. This increase in FGF23 is about the same order of magnitude as the difference in FGF23 that was associated with increased mortality in the CRIC study [9]. In addition, participants only received 6 weeks of sodium bicarbonate in our study, but in clinical practice, patients with CKD are often on sodium bicarbonate therapy for several years. Therefore, the potential effect of sodium bicarbonate on FGF23 could be greater in clinical settings.

There are several explanations for why our findings differ from the *in vitro* data observed by Krieger et al. [13], who found that metabolic acidosis increased FGF23 protein and RNA expression in cultured neonatal mouse calvariae. First, *in vitro* data may differ from *in vivo* due to other unmeasured factors. In an *in vivo* study, Leibrock et al. [18] examined the effect of acidosis induced by ammonium chloride treatment on the phenotype of klotho-hypomorphic mice, which suffer from tissue calcification and reduced life span [19, 20]. FGF23 was significantly higher in these mice compared to wild-type mice, and ammonium chloride treatment significantly decreased intact FGF23 [18]. In clinical studies, Dobre et al. [21, 22] examined the association of serum bicarbonate with cardiovascular morbidity in CKD using the CRIC study and found that high bicarbonate was associated with greater risk of heart failure in multivariable analysis including adjustment for diuretic use. FGF23 induces hypertrophic growth of cardiac myocytes in rodents and promotes left ventricular hypertrophy by activating FGF receptor-4 [23, 24], and left ventricular hypertrophy is an independent risk factor for heart failure [25]. If alkali therapy does indeed increase FGF23, this could contribute to the risk of heart failure in patients with CKD.

Second, the increased FGF23 might not be induced by the alkalinizing effect of sodium bicarbonate as we did not find a statistically significant association between bicarbonate and FGF23 (Table 4). The association between bicarbonate and FGF23 could be falsely negative due to the small sample size and lack of variability in bicarbonate levels because of the entry criteria for the study. Nevertheless, FGF23 could be increased due to other mechanisms not directly related to the alkalinizing effect of sodium bicarbonate: 1) sodium bicarbonate could cause sodium loading; 2) bicarbonate therapy could result in increased protein intake thus higher dietary phosphorus intake and 3) FGF23 might increase with time.

Sodium bicarbonate could increase FGF23 because of the sodium loading. Alkali therapy is usually administered as a sodium or potassium salt. Due to the risk of hyperkalemia in patients with CKD, the sodium salt is often preferred. After 6 weeks of sodium bicarbonate, we found that the mean urinary sodium increased from 2.4 to 2.8 mg/mg Cr (*p* = 0.07) and the result was similar after excluding the 2 participants who had their diuretic dose increased (Table 2). There is no direct evidence on the effect of dietary sodium on circulating FGF23. Sodium loading increases urinary calcium excretion [26–28] as calcium reabsorption depends on the concentration gradient created by the reabsorption of sodium. Increased urinary calcium excretion may decrease serum ionized calcium and stimulate PTH release. PTH can then increase FGF23 by directly stimulating FGF23 gene expression and indirectly via PTH-mediated increase in 1, 25-OH vitamin D [29, 30]. Furthermore, FGF23 has been shown to be involved in renal sodium retention and volume expansion, specifically by regulating the membrane abundance of the NaCl co-transporter, thus increasing distal tubular sodium reabsorption [31]. We speculate that this may provide a mechanistic link between oral sodium bicarbonate, sodium loading and FGF23.

Bicarbonate therapy could increase FGF23 by increasing dietary protein intake. De Brito-Ashurst et al. [32] found that compared to the control group, the bicarbonate group had higher dietary protein intake and albumin level. Increased dietary phosphorus intake from protein may then increase FGF23 [33–35]. However, in our study we found that 6 weeks of sodium bicarbonate therapy decreased urinary nitrogen excretion as previously published [14]. Together with the absence of change in urinary phosphate (Table 2), the speculation that bicarbonate therapy could increase FGF23 by increasing dietary protein intake is blunted.

FGF23 might also increase with time. However, whether FGF23 increases in 6 weeks without any intervention or changes in eGFR is unclear. In a 12-week placebo-controlled study of ferric citrate involving patients with CKD stage 3 to 5 [36], intact FGF23 decreased in the placebo arm from 184 pg/mL [IQR 111–135] to 148 pg/mL [IQR 101–330].

We also accounted for the possible confounding effect of vitamin D. There was a negative association between FGF23 and 1,25-OH vitamin D (Table 4). This is consistent with the data from the CRIC study [9], in which participants with the lowest quartile of FGF23 had the highest 1,25-OH vitamin D level. The relationship between FGF23 and 1, 25-OH vitamin D is a classic negative feedback. While 1, 25-OH vitamin D stimulates FGF23 secretion [37], FGF23 lowers 1,25-OH vitamin D [4]. The use of activated vitamin D has been shown to increase FGF23 in patients with CKD [16, 17]. We performed sensitivity analysis after excluding participants who were taking vitamin D (Table 3). After excluding participants who were taking paricalcitol, the change in FGF23 before and after sodium bicarbonate remained statistically significant. Also, after excluding participants who were taking any form of vitamin D or the participant whose paricalcitol dose was increased, our results were not qualitatively different.

We found that FGF23 was positively associated with serum and urine phosphate (Table 4). This is consistent with the physiological action of FGF23 on phosphate metabolism [10, 35]. FGF23 is associated with high serum phosphate [9], but it is unclear whether high phosphate stimulates FGF23 secretion. Ferrari et al. [35] found that high-phosphate diet increased FGF23 despite stable serum phosphate. Ito et al. [38] showed that acute changes in serum phosphate did not modify FGF23. Infusion of potassium phosphate increased serum phosphate but did not change FGF23 levels. Our findings that FGF23 increased without changes in serum phosphate (Table 2) suggest that the effect of sodium bicarbonate on FGF23 is not mediated by changes in serum phosphate levels.

Our study has several limitations, including the small sample size and lack of a parallel control group. Despite the small sample size, we found significant associations of FGF23 levels with 1,25-OH vitamin D and serum and urine phosphate that were consistent with prior literature and the underlying physiology. Although there was no parallel control group, each participant served as his or her own control by taking placebo for the first 2 weeks. Our findings should also be viewed in the context of the severity of metabolic acidosis in our cohort and the duration of the study. Patients enrolled had relatively mild metabolic acidosis, and the intervention only lasted for 6 weeks. The effect of alkali therapy on bone metabolism might be more pronounced and our result could have been different had we studied individuals with more severe acidosis or had the intervention lasted for a longer period of time. Another limitation is that metabolic acidosis was defined by serum bicarbonate levels without other data regarding acid–base status. This prevents us from fully evaluating the alkalinizing effect of sodium bicarbonate on FGF23. However, this definition of metabolic acidosis was consistent with the National Kidney Foundation Kidney Disease Outcomes Quality Initiative (NKF/KDOQI) guidelines, which suggest treatment of acidosis based on serum bicarbonate alone [39].

## Conclusions

Our pilot study showed that FGF23 levels increased after short-term oral sodium bicarbonate in patients with CKD and mild metabolic acidosis. This might not be due to the alkalinizing effect of sodium bicarbonate, but at the very least, we observed no reduction in FGF23 that would be predicted based on prior *in vitro* work [13]. The NKF/KDOQI suggests maintaining serum bicarbonate ≥ 22 mEq/L in order to retard the effects of metabolic acidosis on progression of CKD and bone loss (Grade 2B) [39–41], and oral sodium bicarbonate is often the preferred choice of alkali therapy. It is critical to elucidate the effect of oral sodium bicarbonate on FGF23 as higher FGF23 has been associated with several adverse outcomes in patients with CKD including cardiovascular mortality. Long-term placebo-controlled studies are needed to substantiate our findings.

## Abbreviations

1,25-OH vitamin D, 1,25-dihydroxyvitamin D; CKD, chronic kidney disease; CRIC, Chronic Renal Insufficiency Cohort; eGFR, estimated glomerular filtration rate; FGF23, fibroblast growth factor 23; IQR, interquartile range; PTH, parathyroid hormone
